# Therapeutic Value of Zinc Supplementation in Acute and Persistent Diarrhea: A Systematic Review

**DOI:** 10.1371/journal.pone.0010386

**Published:** 2010-04-28

**Authors:** Archana Patel, Manju Mamtani, Michael J. Dibley, Neetu Badhoniya, Hemant Kulkarni

**Affiliations:** 1 Lata Medical Research Foundation, Nagpur, India; 2 Indira Gandhi Government Medical College, Nagpur, India; 3 The University of Texas Health Science Center at San Antonio, San Antonio, Texas, United States of America; 4 The Sydney School of Public Health, The University of Sydney, Sydney, Australia; Aga Khan University, Pakistan

## Abstract

**Background:**

For over a decade, the importance of zinc in the treatment of acute and persistent diarrhea has been recognized. In spite of recently published reviews, there remain several unanswered questions about the role of zinc supplementation in childhood diarrhea in the developing countries. Our study aimed to assess the therapeutic benefits of zinc supplementation in the treatment of acute or persistent diarrhea in children, and to examine the causes of any heterogeneity of response to zinc supplementation.

**Methods and Findings:**

EMBASE®, MEDLINE ® and CINAHL® databases were searched for published reviews and meta-analyses on the use of zinc supplementation for the prevention and treatment of childhood diarrhea. Additional RCTs published following the meta-analyses were also sought. The reviews and published RCTs were qualitatively mapped followed by updated random-effects meta-analyses, subgroup meta-analyses and meta-regression to quantify and characterize the role of zinc supplementation with diarrhea-related outcomes. We found that although there was evidence to support the use of zinc to treat diarrhea in children, there was significant unexplained heterogeneity across the studies for the effect of zinc supplementation in reducing important diarrhea outcomes. Zinc supplementation reduced the mean duration of diarrhea by 19.7% but had no effect on stool frequency or stool output, and increased the risk of vomiting. Our subgroup meta-analyses and meta-regression showed that age, stunting, breast-feeding and baseline zinc levels could not explain the heterogeneity associated with differential reduction in the mean diarrheal duration. However, the baseline zinc levels may not be representative of the existing zinc deficiency state.

**Conclusions:**

Understanding the predictors of zinc efficacy including the role of diarrheal disease etiology on the response to zinc would help to identify the populations most likely to benefit from supplementation. To improve the programmatic use of zinc, further evaluations of the zinc salts used, the dose, the frequency and duration of supplementation, and its acceptability are required. The significant heterogeneity of responses to zinc suggests the need to revisit the strategy of universal zinc supplementation in the treatment children with acute diarrhea in developing countries.

## Introduction

Despite significant improvements in the interventions to treat diarrhea in children, it continues to pose a daunting public health challenge, especially in children from developing countries. Recent estimates suggest that nearly 3% of neonatal mortality and 17% of under-five child mortality is attributable to diarrhea. Asia and Africa have an alarmingly high incidence of childhood diarrhea. [1], [2], [3] Although the burden of the diarrhea-related mortality has significantly decreased since the introduction of oral rehydration therapy in 1980, diarrheal diseases in children remain a substantial global health problem. [4], [5], [6] In 2004, the World Health Organization (WHO) and the United Nations Children's Fund (UNICEF) took two significant steps to reduce this burden by recommending the use of low-osmolarity oral rehydration solution (ORS), and supplementation with zinc for up to two weeks as part of the case management of acute diarrhea. [7], [8]

The latter recommendation was based on the results of several randomized controlled trials, meta-analyses [9] and reviews [10], [11], [12], [13] reported from around the world that have demonstrated the utility of zinc supplementation to shorten the duration of diarrhea and improve other diarrhea related outcomes. Nearly five years have elapsed and substantial additional evidence [14], [15], [16], [17], [18], [19] has accumulated since the inception of the practice of zinc supplementation. The existing paradigm strongly supports the notion of zinc supplementation; however, recent scientific reports suggest several interesting cues described below indicate that a more focused approach to zinc supplementation may be required.

First, WHO/UNICEF recommends zinc supplementation for diarrhea in developing countries only. [19] The underlying justification for this is the differential prevalence of zinc deficiency. Extension of this line of thought would suggest that differential levels of zinc deficiency in individuals or populations within developing countries might modulate the therapeutic benefits attributable to zinc. Second, five meta-analyses have been published thus far [9], [16], [17], [18], [20], [21]that have all observed a protective effect of zinc on some diarrhea outcomes, but all of these meta-analyses have also reported a significant degree of heterogeneity in effect sizes across studies. Such heterogeneity raises concerns regarding the reliability of the synthetic estimates of the use of zinc supplementation. Third, evidence is emerging that zinc supplementation is not equally effective against all causative organisms. [22], [23] Since the causes of acute diarrhea even within developing countries vary widely, the efficacy of zinc supplementation is likely to be heterogeneous. Lastly, it is not clear at present how zinc supplementation complements, if at all, other possible options like vitamin A supplementation and multivitamin supplementation. [23], [24], [25]

Together, these issues indicate the need for a closer look at the evidence that underpins the policy of blanket zinc supplementation to children with diarrhea in developing countries. This study aimed to assess the therapeutic benefits of zinc supplementation in the treatment of acute or persistent diarrhea in children, and to examine the causes of any heterogeneity of response to zinc supplementation.

## Methods

### Data Extraction

**Figure 1:**
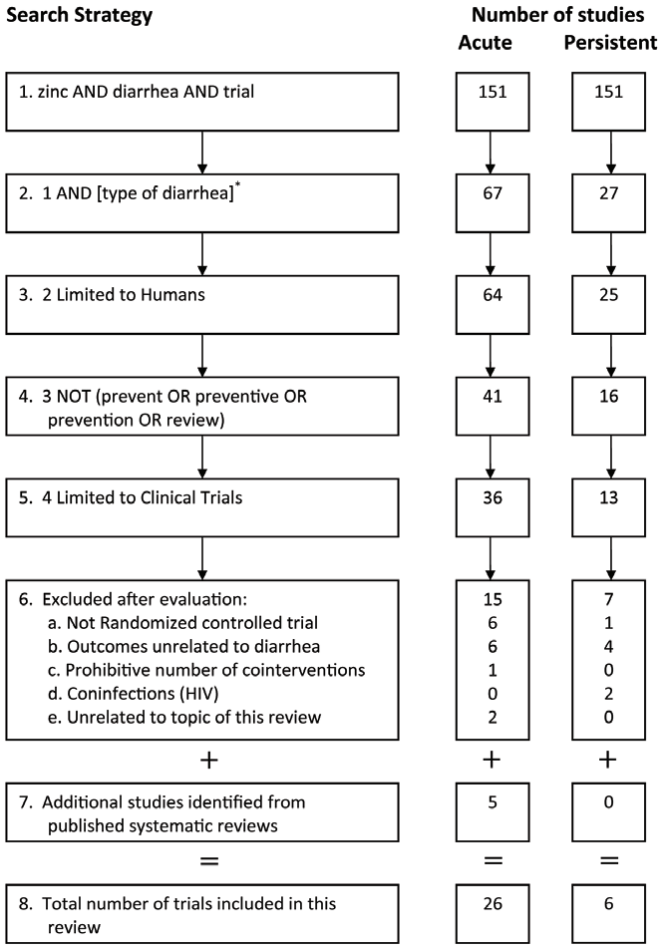
Flowchart for study selection protocol in the present study. *, Type of diarrhea was included as acute or persistent.

Data extraction for this study was conducted in two steps. First, we searched the EMBASE®, CINAHL® and MEDLINE® databases for published trials on zinc supplementation. The full strategy for searching these databases and the results obtained are shown in Figure 1. Second, we collected the published reviews and meta-analyses in this field. For this, we searched the same databases using the query “zinc AND diarrhea” and limiting the citations to reviews, we identified 129 review articles of which 50 dealt with “zinc supplementation”. Further restricting the articles to publication type “meta-analysis” identified 10 articles of which seven had formally conducted synthesis of published trials on the preventive or the therapeutic role of zinc in acute or persistent diarrhea. Five of these seven meta-analyses related to the therapeutic use of zinc in diarrhea. We carefully reviewed these five meta-analyses for any additional studies that we may have missed in the first stage of the search (Figure 1, step 7). In total, we identified 26 trials for acute diarrhea and 6 trials for persistent diarrhea. Attached at the end of the manuscript are the PRISMA statement and flowchart detailing the methods of data extraction and abstraction.

### Analytical approach

We constructed a correspondence map of the published studies and meta-analyses to identify which studies were included in the different meta-analyses. We then summarized the findings from these meta-analyses into diarrhea-related clinical end-points. For each outcome, we examined the reported summary effect sizes and the heterogeneity across studies. For quantifying heterogeneity, we used the I^2^ statistic since it is comparable across meta-analyses. [26] If a meta-analysis reported the Q test result for heterogeneity then the I^2^ statistic was estimated from it using the formula I^2^ = (Q-df)/Q with the minimum bound set to zero.

For major outcomes that showed significant summary beneficial effect of zinc on diarrhea, and which showed large heterogeneity across trials, we investigated the potential contributors to the heterogeneity. First, we conducted an updated meta-analysis to include the results from other studies that the previous meta-analyses may have omitted. For these meta-analyses, we used the random effects model of DerSimonian and Laird. [27] Depending on the diarrhea related outcome, we used standardized mean difference or summary odds ratios as the summary measures for effect size. For diarrhea-related outcomes showing substantial heterogeneity across studies, we then estimated the contribution of potential predictors of effect size to between-study heterogeneity. For predictor variables that were categorical in nature (geographic location and setting of the study, zinc salt used, co-intervention used, and adequacy of blinding procedures) we used subgroup meta-analyses. For continuous predictor variables we conducted univariate meta-regression analyses as recommended by Higgins et al [26] and Thomson et al [28]. Continuous variables included in these analyses were: mean age, dose of zinc, duration of diarrhea before admission, proportion wasted defined as weight-for-age z score<-2, proportion stunted defined as height-for-age z score<-2, mid-arm circumference, proportion with fever, mean dehydration score, baseline zinc and proportion breastfed. Statistical analyses were conducted using the Stata 10.2 (Stata Corp, College Station, TX) software package.

## Results

### Systematic map of published meta-analyses on zinc supplementation in diarrhea

In 1998, Black et al [10] conducted the first focused literature review of zinc supplementation, which provided a significant impetus for the formulation of the WHO/UNICEF recommendation [7] six years later on the use of zinc in the treatment of childhood diarrhea. Together the published meta-analyses have summarized data from 23 randomized controlled trials (9,958 children receiving zinc and 9,940 subjects receiving placebo) excluding four studies [29], [30], [31], [32] published after the meta-analyses. For ease of identification, these meta-analyses are labeled chronologically as M1–M5 in Table 1.

**Table 1:** Published studies therapeutic use of zinc against in acute diarrhea.

No	Author [Ref]	Year	Zn	Pl	M1	M2	M3	M4	M5
1	Sachdev et al [57]	1990	20	20		X		X	
2	Sazawal et al [47]	1995	456	481	X	X	X	X	X
3	Roy et al [45]	1997	37	37	X	X	X		X
4	Hidayat et al [42]	1998	738	659	X	X	X		X
5	Roy et al [53]	1998	95	95	X			X	
6	Faruque et al [39]	1999	341	340		X	X	X	
7	Dutta et al [38]	2000	44	36		X	X	X	X
8	Khatun et al [50]	2001	44	44				X	
9	Strand et al [48]	2002	442	449		X	X	X	
10	Bahl et al [34]	2002	806	401		X	X		X
11	Baqui et al [66]	2002	3974	4096		X			
12	Al-Sonboli et al [33]	2003	37	37		X	X	X	X
13	Polat et al [44]	2003	92	90		X	X	X	X
14	Bhatnagar et al [35]	2004	132	134		X	X	X	X
15	Brooks et al [37]	2005	171	89		X	X	X	X
16	Larson et al [67]	2005	534	533			X	X	
17	Patel et al [43]	2005	102	98		X			X
18	Valery et al [25]	2005	107	108		X			
19	Fischer Walker et al [40]	2006	538	536		X	X	X	X
20	Awasthi et al [49]	2006	1010	992			X		
21	Boran et al [36]	2006	150	130			X		X
22	Roy et al [52]	2007	28	28			X	X	
23	Gregorio et al [41]	2007	60	57			X		X
24	Roy et al [32]	2008	82	82				X	
25	Patel et al [31]	2009	535	273					
26	Fijolu et al [29]	2009	30	30					

X indicates that the trial was included in the specified meta-analysis.

M1, Bhutta et al 2000 [9]; M2, Lukacik et al 2008 [17]; M3, Patro et al 2008 [18]; M4, Lazzerini et al 2008 [16]; M5, Haider and Bhutta [15].

Zn, Lumber of subjects in the Zn supplementation Group; Pl, Number of subjects in the placebo Group.

The RCTs of the therapeutic effects of zinc supplementation during diarrhea have reported a wide variety of diarrhea-related outcomes. For example, these RCTs report the domain of diarrheal duration in various ways as mean duration of diarrhea since initiation of treatment, the percentage reduction in the duration of diarrhea, and the proportion of children with continued diarrhea beyond a predefined number of days (1, 3, 5 or 7). In addition, other outcomes have included stool frequency, stool output, risk of vomiting and risk of watery stools. All the five meta-analyses [9], [15], [16], [17], [18] and most of the published randomized controlled trials have however, reported the effect of zinc supplementation on mean diarrheal duration. Two meta-analyses [9], [17], report that there is about 15–16% reduction in the mean duration of acute diarrhea while four meta-analyses [15], [16], [17], [18] report that zinc supplementation can reduce the acute diarrheal duration by 0.24 to 0.67 days (Table 2). Again, for this important outcome, the more recent meta-analyses [15], [16], [17], [18] suggested that the published evidence demonstrates a statistically significant degree of heterogeneity with I^2^ statistic ranging from 73% to 85% (Table 2). Alternatively expressed, zinc supplementation appears to reduce the risk of continued diarrhea beyond 7 days by 29% (Table 2), although the results were heterogeneous across the published literature (I^2^>70%). On the other hand, zinc supplementation does not provide a statistically significant reduction in stool frequency or stool output and this evidence was not heterogeneous (Table 2).

**Table 2:** Outcomes and summary effects related to acute diarrhea observed in published meta-analyses.

Outcome	Meta-analysis	RCTs	N	Statistic	ES	95% CI	I^2^ (%), p
Recovery from diarrhea	M1	3	2446	RH	0.85*	0.76–0.95	65, 0.04
Diarrhea at day 1	M2	5	3100	RR	1.01	0.99–1.03	63, 0.03
Diarrhea at day 3	M2	6	3908	RR	0.97	0.91–1.03	55, 0.05
	M3	3	1630	RR	0.62*	0.44–0.87	---
	M4	3	1073	RR	0.69*	0.59–0.81	48, 0.116
Diarrhea at day 5	M2	6	3908	RR	0.94	0.84–1.05	74, 0.002
	M3	2	346	RR	0.68	0.11–4.31	---
	M4	2	346	RR	0.55*	0.32–0.95	43, 0.19
Diarrhea for ≥7 days	M1	3	289	OR	0.78	0.56–1.09	0, 0.71
	M3	8	5769	RR	0.71*	0.53–0.96	---
	M4	10	4087	RR	0.71*	0.52–0.98	73, 0.0001
Duration of diarrhea	M1	5	3177	% ↓	16.2*	6.8–25.6	0, 0.56
	M2	16	15272	% ↓	15.0	---	84, 7.5×10^−14^
	M2	16	15272	WMD, d	0.24*	0.21–0.27	84.3, 8.9×10^−14^
	M3	13	5643	WMD, d	−0.69*	−0.97–−0.40	73, 1.6×10^−6^
	M4	13	2741	WMD, h	−12.27*	−23.02–−1.52	85, 1.8×10^−11^
	M5	14	5670	WMD, d	−0.50*	−0.82–−0.08	84, 4.1×10^−12^
Stool frequency	M2	7	3117	% ↓	18.0	---	---
	M3	3	1384	WMD	−0.02	−0.29–0.25	---
	M4	7	1458	WMD	−0.02	−0.19–0.15	53, 0.05
Stool output	M2	3	478	% ↓	30.3	---	---
	M3	3	606	WMD	−0.38	−1.04–0.27	---
Vomiting	M2	11	4438	RR	1.55*	1.30–1.84	60.8, 0.004
	M3	5	3156	RR	1.22*	1.05–1.43	---
	M4	10	4727	RR	1.71*	1.27–2.30	69.3, 0.001
Watery stools	M3	3	3476	RR	0.86*	0.77–0.97	---

M1, Bhutta et al 2000 [9]; M2, Lukacik et al 2008 [17]; M3, Patro et al 2008 [18]; M4, Lazzerini et al 2008 [16]; M5, Haider and Bhutta [15].

OR, odds ratio; RR, relative risk; RH, relative hazards; WMD, weighted mean difference; RCT, Lumber of randomized control trials used; N, Number of subjects included in meta-analysis; ES, summary effect size, CI, confidence interval; d, days; h, hours; % ↓, percentage reduction.

M1 reported Q statistic and degrees of freedom and the I^2^ statstic was derived using the formula I^2^ = (Q-df)/Q. *, statistically significant; ---, not mentioned and not estimable.

Meta-analytical synthesis of the influence of zinc supplementation is available for three more outcomes: persistent diarrhea, vomiting after zinc administration and childhood mortality. A smaller number of trials provide the current evidence for the effects of zinc supplementation on persistent diarrhea compared to acute diarrhea. Nonetheless, zinc supplementation offers a clear benefit for persistent diarrhea and this effect was homogeneous across the published studies (Table 3). Three meta-analyses [16], [17], [18] have summarized the results from randomized controlled trials with vomiting as an outcome, all of which found that the risk of vomiting significantly increased after zinc supplementation [point estimates for odds ratios (OR) ranging from 1.22 to 1.71, Table 2]. The most recent meta-analysis reported significant heterogeneity across study results (I^2^ 69%, Table 2).

**Table 3:** Outcomes and summary effects related to persistent diarrhea observed in published meta-analyses.

Outcome	Meta-analysis	RCTs	N	Statistic	ES	95% CI	I^2^ (%), p
Recovery from persistent diarrhea	M1	4	680	RH	0.76*	0.63–0.91	---
Occurrence of diarrhea at day 1	M2	2	221	RR	1.00	0.93–1.08	0, 0.93
Occurrence of diarrhea at day 3	M2	2	221	RR	0.70*	0.51–0.94	0, 0.56
Continuation of diarrhea >7 days	M1	4	680	RR	0.61	0.26–1.46	---
Duration of persistent diarrhea	M1	4	680	% ↓	29.3*	6.0–52.5	0, 0.559
	M2	5	489	% ↓	15.5	---	---
	M2	5	489	WMD, d	0.299*	0.120–0.478	29.9, 0.544
Vomiting							
	M2	4	2969	RR	3.64*	1.02–13.02	49.2, 0.116

M1, Bhutta et al 2000 [9]; M2, Lukacik et al 2008 [17].

OR, odds ratio; RR, relative risk; RH, relative hazards; WMD, weighted mean difference; RCT, Lumber of randomized control trials used; N, Number of subjects included in meta-analysis; ES, summary effect size, CI, confidence interval; d, days; % ↓, percentage reduction.

M1 reported Q statistic and degrees of freedom and the I^2^ statstic was derived using the formula I^2^ = (Q-df)/Q. *, statistically significant; ---, not mentioned and not estimable.

### Investigation into the heterogeneity: meta-analyses and meta-regressions

The systematic map shows that accompanying the influence of zinc supplementation on diarrhea was heterogeneity of the results across the published trials. We therefore conducted an investigation into the potential contributors to this heterogeneity. Summarizing results from Tables 1 and 2, we focused our analyses on two outcomes: mean duration of diarrhea in therapeutic trials and the risk of vomiting in therapeutic trials. For each of these outcomes we first implemented a random effects meta-analysis and then undertook subgroup meta-analysis and meta-regression.

#### Zinc supplementation and mean diarrheal duration from therapeutic trials

**Figure 2:**
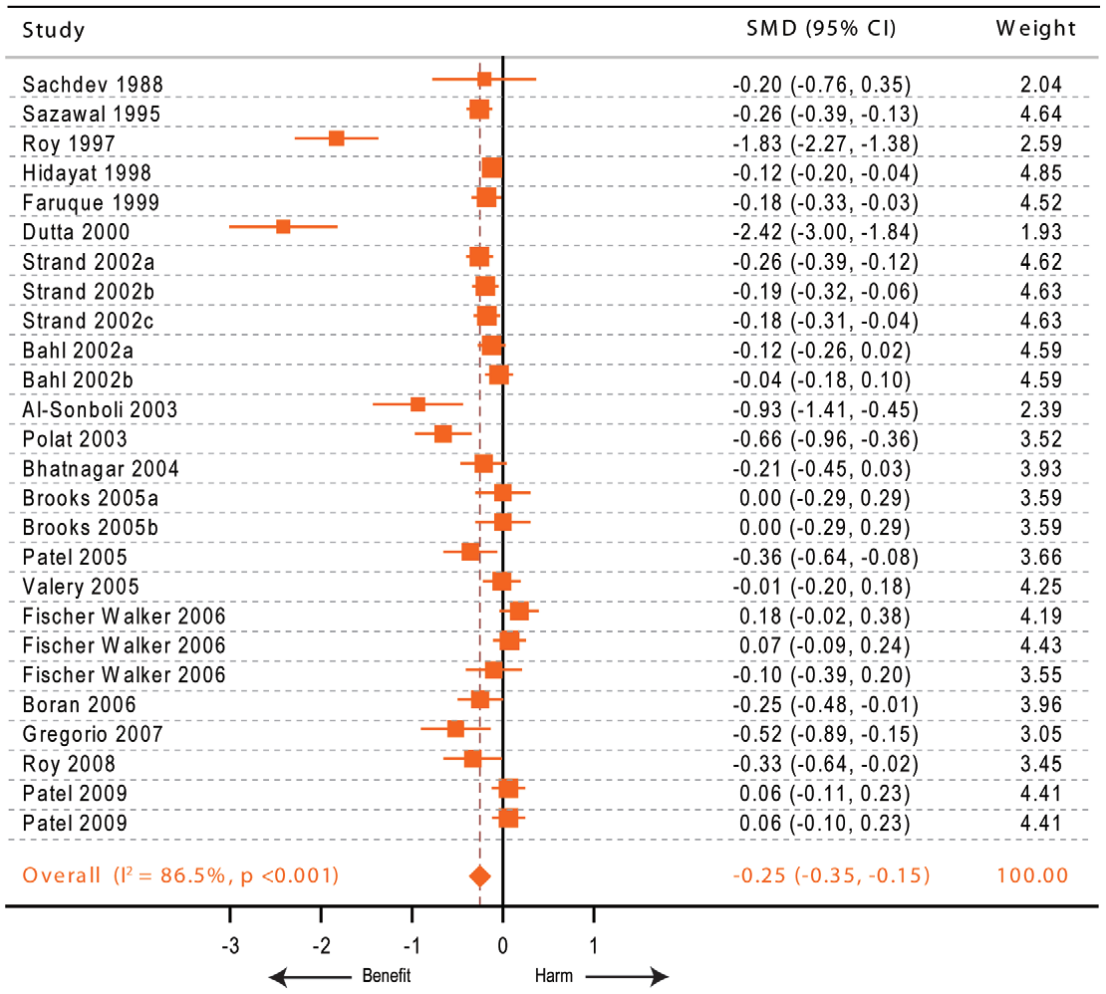
Forest plot depicting the studies included in our meta-analysis for the outcome of mean diarrheal duration. Orange squares and lines indicate the point and 95% confidence intervals for the standardized mean deviations (SMD) and the orange diamond denotes the point and confidence interval for the summary effect size. Suffixes a, b and c indicate specific zinc-treated subgroups within the indicated study. Weights are expressed in percentage.

Our updated meta-analysis for this outcome (Figure 2) showed that the published data come off 26 comparisons from 19 trials [25], [31], [32], [33], [34], [35], [36], [37], [38], [39], [40], [41], [42], [43], [44], [45], [46], [47], [48] representing 8,957 children. We excluded trials that either studied the effects of zinc supplementation on future episodes or did not report the mean duration (and measures of variability) of the current diarrheal episodes [29], [49], [50], [51], [52], [53]. Our results support a statistically significant effect of zinc supplementation on mean diarrheal duration [standardized mean difference (SMD) −0.25, 95% CI −0.35–−0.15]. Considering the statistical properties of SMD [54], this translates into a reduction in mean diarrheal duration by 19.7% (95% CI 11.9%–27.4%). The extent of heterogeneity across studies was statistically significant (I^2^ 86.5%, p<0.001). For this outcome our subgroup meta-analyses (Table 4) showed that the country of origin could not explain the heterogeneity, however age <12 months and study setting were associated with a differential reduction in the mean diarrheal duration. We also observed that the beneficial effect of zinc was influenced by studies that recruited all the study subjects before 12 months of age. We observed that in the five study groups from two studies that recruited infants only the SMD was 0.06 whereas when analysis was restricted to the studies that included other age groups also the SMD was −0.32 – a difference that was highly statistically significant (unpaired Student's t test p-value for difference in SMDs = 0.006). The hospital-based studies [25], [31], [32], [33], [35], [37], [38], [39], [43], [45], [46] were more likely to show improvement as compared to studies conducted in community settings [34], [40], [41], [42], [47], [48] (SMD −0.33 versus −0.13, respectively and unpaired Student's t test p value = 0.049). Studies using zinc gluconate [34], [47], [48] and those using vitamin A as a co-intervention [25], [39], [48] showed a significant reduction in diarrheal duration and were homogeneous (Table 4).

**Table 4:** Results of subgroup meta-analyses for the outcomes of mean diarrheal duration and risk of vomiting.

Variable/Category			Duration				Vomiting	
	SG*	SMD	95% CI	I^2^	SG*	OR	95% CI	I^2^
Location								
India	10	−0.23	−0.42–−0.03	89.6	5	1.19	0.87–1.64	43.0
Bangladesh	5	−0.44	−0.89–0.02	92.8	2	2.18	0.91–5.22	0.0
Indonesia	1	−0.12	−0.20–−0.04	---				
Nepal	3	−0.21	−0.28–−0.13	0.0	3	4.23	3.26–5.49	0.0
Brazil	1	−0.93	−1.41–−0.45	---				
Turkey	2	−0.44	−0.84–−0.04	77.5	2	6.18	1.92–19.9	0.0
Australia	1	−0.01	−0.20–0.18	---	1	0.51	0.05–5.71	---
Pakistan	1	0.07	−0.09–0.24	---				
Ethiopia	1	−0.10	−0.39–0.20	---				
Philippines	1	−0.52	−0.89–−0.15	---				
Age ≥12m								
Yes	21	0.06	−0.04–0.16	0.0	10	2.23	1.29–3.85	85.2
No	5	−0.32	−0.44–−0.21	87.7	3	1.59	1.37–3.31	0.0
Setting								
Hospital	13	−0.42	−0.67–−0.18	91.6	4	1.19	0.65–2.20	24.1
Community	11	−0.13	−0.20–0.05	65.7	7	2.26	1.33–3.84	87.0
Unclear	2	−0.44	−0.84–−0.04	77.5	2	6.18	1.92–19.9	0.00
Zinc salt								
Acetate	6	−0.35	−0.64–−0.07	91.5	2	2.18	0.91–5.22	0.0
Gluconate	6	−0.18	−0.24–−0.11	30.0	6	2.44	1.33–4.47	88.0
Sulfate	14	−0.31	−0.51–−0.11	88.8	5	1.58	0.78–3.21	61.4
Co-intervention								
None	14	−0.16	−0.26–−0.07	72.5	8	2.55	1.40–4.63	82.8
Vitamin A	3	−0.15	−0.25–−0.05	21.1	2	2.07	0.30–14.4	64.3
Multivitamins	2	−1.01	−2.59–0.58	97.5	2	0.87	0.52–1.44	0.0
Erythromycin	1	−0.33	−0.64–−0.02	---				
ORS	6	−0.43	−0.79–−0.08	93.5	1	1.74	1.14–2.66	---
Efficient blinding								
Yes	17	−0.24	−0.36–−0.15	89.0	9	2.01	1.26–3.21	84.8
Unclear	7	−0.29	−0.51–0.07	75.2	3	3.28	0.67–16.1	42.0

*, Number of study groups included in meta-analysis.

**Figure 3:**
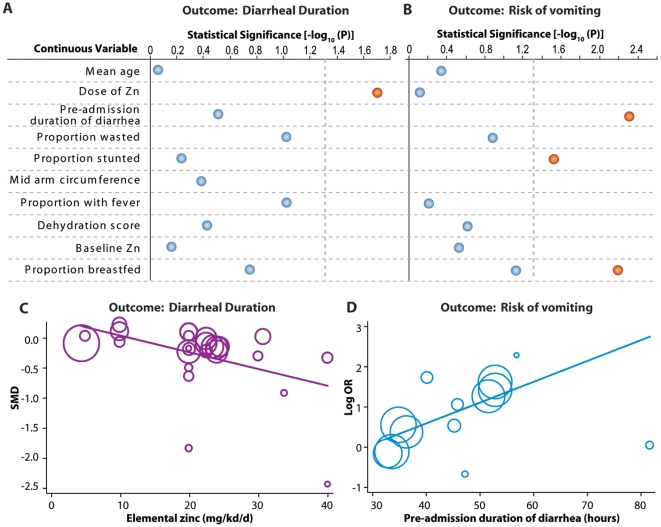
Investigation of the potential contribution of continuous variables to heterogeneity across study results for the outcomes of mean diarrheal duration and risk of vomiting. (**A and B**) Results from univariate meta-regression for continuous variables as predictors of the between-study heterogeneity for mean diarrheal duration (A) and risk of vomiting (B). The statistical significance is shown as log transformed p-value and the vertical dashed line corresponds to a p-value of 0.05. Blue dots, statistically insignificant; red dot, statistically significant. (**C and D**) Bubble plots showing the influence of the dose of elemental zinc as a predictor of the standardized mean difference of diarrheal duration (C) and log odds ratio of the risk of vomiting (D). Each bubble represents a study group listed in Figure 2 and the size of the bubble is proportional to the inverse-variance weights.

We also explored the effect of causative organisms. Seven trials [31], [32], [33], [35], [38], [45], [46] have reported the array of causative organisms for diarrhea and in the present review we observed that the effect of zinc on mean diarrheal duration was significant in trials not reporting *Esherichia coli* and rotavirus as the causes (SMD −0.14, 95% CI −0.21–−0.07; data not shown). Finally, results of our meta-regression analyses showed (Figure 3a and c) that the dose of zinc was the only variable that was statistically significantly associated with diarrheal duration – trials using higher doses generally reported larger effect of zinc supplementation on mean diarrheal duration (p = 0.02). Interestingly, average baseline zinc levels did not contribute to between-study variations in the effect size (p = 0.70)

#### Zinc supplementation and risk of vomiting

**Figure 4:**
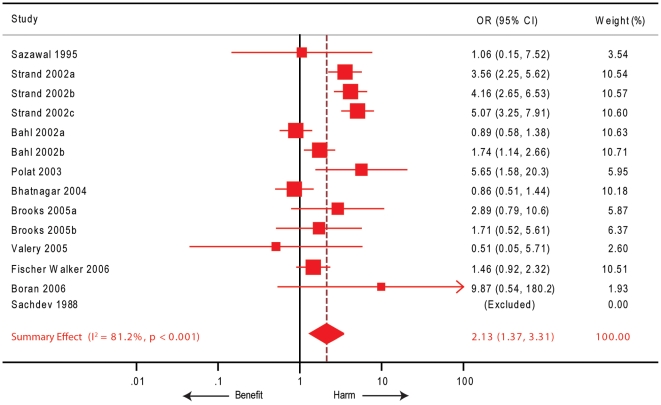
Forest plot depicting the studies included in our meta-analysis for the outcome of risk of vomiting. Red squares and lines indicate the point and 95% confidence intervals for the odds ratios (OR) and the red diamond denotes the point and confidence interval for the summary effect size. Suffixes a, b and c indicate specific zinc-treated subgroups within the indicated study. Weights are expressed in percentage.

Rates of vomiting after zinc administration have been reported in 14 comparisons from 10 trials [25], [34], [35], [36], [37], [40], [44], [46], [47], [48] representing 6,779 children. In a quantitative synthesis of these results (Figure 4), we observed that the risk of vomiting was significantly increased after zinc administration (19.2% in the zinc supplemented group and 9.2% in the zinc withheld group) summary OR 2.13, 95% CI 1.37–3.31). However, this zinc effect was significantly heterogeneously distributed across the trials (I^2^ 81.2%, p<0.001).

In the subgroup analyses (Table 4) we found that studies from India [34], [35], [40], [47], studies using zinc acetate [37], those using multivitamins as a co-intervention [35], [47], and those in which the efficiency of the blinding procedure was unclear [25], [36], [44] were homogeneous in terms of the reported associations. Of these subgroups, the studies from India, studies using zinc acetate and those using multivitamins did not show a significant association of zinc supplementation with vomiting. The strongest association with vomiting was found in studies [34], [36], [37], [40], [44], [48] that used no co-intervention in addition to zinc (OR 2.55, 95% CI 1.40–4.63). In addition, well-blinded studies [34], [35], [37], [40], [47], [48], those studies conducted in hospital settings [25], [35], [37] and studies using zinc gluconate [34], [47], [48] reported a high degree of association between zinc supplementation and the risk of vomiting. In meta-regression analyses (Figure 3b and d), we observed that the duration of diarrhea before admission (13 comparison groups, p = 0.005), the proportion of the children who were stunted (8 comparison groups, p = 0.027) and the proportion of children who were breastfed (6 comparison groups, p = 0.006) were the variables that were significantly associated with the reported rates of vomiting. Since the inference for the association of pre-admission diarrheal duration with vomiting came from almost all the studies included in this meta-regression, we specifically examined this association. The I^2^ statistic after accounting for pre-admission diarrheal duration shrunk from 81.2% to 48.1% indicating that a large proportion of the variability across trials could be explained by the duration of pre-admission diarrhea.

### Influence of zinc supplementation on persistent diarrhea

Table 3 demonstrates that zinc supplementation has a clear benefit in reducing the incidence of persistent diarrhea by approximately 25%. It improved the recovery from persistent diarrhea by 24% and reduced the proportion of children with persistent diarrhea extending beyond three days after zinc supplementation by 30%. It also reduced the mean duration of persistent diarrhea by 21.5–29.3%, although it was associated with a significantly high risk of vomiting. For all these outcomes, the existing evidence demonstrates a high degree of homogeneity of effects across the published trials. The most recent meta-analysis [16] reported on five trials in children with persistent diarrhea [50], [53], [55], [56], [57]. Three trials reported on diarrhea at day three [44], [48], [56], three trials on diarrhea at day five [35], [38], [56] and nine at day seven [35], [39], [40], [44], [47], [48], [50], [52], [56]. There was a reduction in persistent diarrhea by −15.84% [95% CI −25.43–−6.24%]. As no new trials on influence of zinc on outcomes of persistent diarrhea have been published since the publication of these meta-analyses, we did not conduct a redundant synthetic investigation into this effect of zinc. Also, due to the small number of trials we did not conduct subgroup analyses or meta-regression for the outcome of persistent diarrhea.

## Discussion

The results of our systematic review suggest that zinc supplementation reduced the mean duration of acute diarrhea by approximately 20%, and persistent diarrhea by 15–30%, but had no significant effect on stool frequency or stool output. Further it was associated with a two- to three-fold higher risk of regurgitation in acute and persistent diarrhea, respectively. There was a high degree of statistically significant heterogeneity across the published studies for the effects of zinc supplementation on mean diarrheal duration and risk of vomiting following the administration of zinc.

Consistent with the existing understanding [15], [58], the therapeutic trials showed that zinc would reduce diarrhea by nearly a day for an average episode of five days, but again there was a high degree of heterogeneity of this effect across the published studies. The World Health Organization (WHO) recommends zinc supplementation (10–20mg for 10–14 days) for treatment of acute diarrhea. [7] Although the recommendation does not specify the salt, our subgroup analysis showed a significant homogeneous reduction in diarrheal duration in studies using zinc gluconate [34], [48] and those using vitamin A as a co-intervention [25], [39], [48] (Table 4). Vitamin A supplementation up-regulates the Th2 immune response, while zinc supplementation up regulates Th1 responses, and perhaps these interventions have synergistic effects, a research question which needs further exploration. [23]

Interestingly, higher doses of zinc in the therapeutic trials were associated with larger reductions in the mean duration of diarrhea. It is difficult to comment on the potential relationship between zinc dose and diarrheal duration in the context of achieving a balance between the reduction of diarrhea and risk of vomiting. If similar benefits of treatment are possible with lower doses and there is a lower risk of vomiting with these doses then lower doses might be advisable. Specific dose-response trials to examine this question would therefore be more appropriate. Similarly, the fact that subgroup analysis but not meta-regression demonstrated a differential benefit of zinc supplementation indicates that there may be a threshold for age beyond which zinc supplementation may be useful. However, the lack of an association between mean age in a trial and the effect of zinc may also reflect a lack of informative content in mean age as a contributor to heterogeneity and thus trials focused to address these issues would be appropriate.

An important finding from our analyses was that zinc supplementation showed no effect on stool frequency and output. This opens up the possibility that care-givers may not perceive a beneficial impact of treating their children with zinc, which might negatively affect their adherence to the treatment regime. Another potential barrier to treatment adherence with zinc supplements was the significantly increased risk of vomiting. These findings imply that use of zinc supplements is unlikely to improve compliance with the treatment for diarrheal disease. It is noteworthy however, that a recent study of the safety of zinc supplementation in acute diarrhea suggests that most of the patients regurgitate only once – a phenomenon that may not affect the continuation of zinc therapy. [59] Further, in our subgroup analyses, compared to zinc acetate, zinc gluconate was significantly associated with a reduction in the duration of diarrhea but also showed a significantly increased risk of vomiting. These findings highlight the imbroglio facing those designing an intervention program - which salt should be used, at what dose, frequency, and duration, to maximize the acceptance and benefits of zinc supplementation as an adjunct in the treatment of childhood diarrhea. Moreover, the fact that zinc supplementation was beneficial in the absence of *E. coli* and rotavirus calls for a closer look into the clinical and public health scenarios where this intervention may be most beneficial. Whether such a strategy of zinc supplementation that is tiered on the basis of the causative organism will be efficacious and effective is currently unknown. The WHO recommendations do not fully address these issues and protocols for the use of zinc in diarrhea treatment programs need revisiting.

Stunted children are likely to be zinc deficient and therefore should benefit from zinc supplementation [60], [61]. Our analysis surprisingly showed that factors such as age, malnutrition (proportion stunted and wasted), breast feeding, dehydration at enrolment and baseline zinc, which have previously been reported to affect the response to zinc for reducing duration of diarrhea, did not show a statistically significant effect. It should be noted, however, that the baseline plasma zinc concentrations reported in trials may not fully capture the state of zinc deficiency in the children. Such values are likely confounded by acute phase response, diurnal variations and time since previous meals [62], [63], [64]. Therefore, our observed lack of an association between baseline plasma concentration and zinc efficacy should be cautiously interpreted. On the other hand diarrheal duration before admission, stunting, wasting, and being breast fed were factors significantly associated with the reported rates of vomiting following supplementation with zinc. Thus, the population most likely to benefit from zinc supplementation was also the population at an increased risk of vomiting.

Meta-analyses and meta-regression, like any study design, have inherent limitations. For example, because only a few studies reported information on most of the predictor variables simultaneously, we could not conduct multivariate meta-regression analyses. Thus, the importance of the factors that we identified is unknown in a multivariate context. In addition, even though meta-regression can provide important clues into the potential contributors to the summary effect size [65], it can only examine those covariates reported in the trials examined. Only eight trials [24], [31], [32], [33], [34], [38], [45], [46] have reported as covariates, diarrheal etiology or causative organisms as covariates. In the present review we observed that the effect of zinc on mean diarrheal duration was significant in trials where *Esherichia coli* and rotavirus were not causes of the diarrhea (SMD −0.14, 95% CI −0.21–−0.07). The role of other unknown covariates like adherence and acceptability remains unknown. Indeed, we did observe that significant heterogeneity across study results remained even after accounting for the potential predictors of between-study heterogeneity. Lastly, subgroup analyses and meta-regression are, by disposition, exploratory tools to provide pointers towards possible sources of heterogeneity - they cannot be taken as confirmatory tests for definitive conclusions and interpretations about the causes of heterogeneity.

Our findings for the use of zinc in the treatment of diarrhea indicate the need to improve upon the current strategy of zinc supplementation for all children with diarrhea, by selecting the populations most likely to benefit from supplementation and using the most effective zinc salt. There is a need to optimize the use of zinc supplementation in childhood diarrheas and this will require further investigation of the factors leading to the heterogeneity of the effects of zinc as an adjunct in its treatment.
